# Antibacterial Activity of* Salvadora persica* L. (Miswak) Extracts against Multidrug Resistant Bacterial Clinical Isolates

**DOI:** 10.1155/2016/7083964

**Published:** 2016-01-19

**Authors:** Mohamed Saeed Zayed Al-Ayed, Ahmed Morad Asaad, Mohamed Ansar Qureshi, Hany Goda Attia, Abduljabbar Hadi AlMarrani

**Affiliations:** ^1^Department of Pediatrics, College of Medicine, Najran University, P.O. Box 1988, Najran, Saudi Arabia; ^2^Microbiology Department, College of Medicine, Najran University, P.O. Box 1988, Najran, Saudi Arabia; ^3^Pharmacognosy Department, College of Pharmacy, Najran University, P.O. Box 1988, Najran, Saudi Arabia; ^4^The Faculty of Sharia and Fundamentals of Religion, Najran University, P.O. Box 1988, Najran, Saudi Arabia

## Abstract

Much effort has focused on examining the inhibitory effect of* Salvadora persica* (miswak) on oral microorganisms, but information concerning its antibacterial activity against other human pathogens, particularly multidrug resistant (MDR) isolates, is scarce. Therefore, this study aimed to assess the in vitro antibacterial activities of* Salvadora persica* L. extracts against 10 MDR bacterial clinical isolates other than oral pathogens. The antibacterial activity of aqueous and methanol miswak extracts was assessed using the agar dilution and minimum inhibitory concentration (MIC) methods. Overall, the 400 mg/mL of miswak extract was the most effective on all strains. The methanol extract exhibited a stronger antibacterial activity against Gram-negative (3.3–13.6 mm) than Gram-positive (1.8–8.3 mm) bacteria. The lowest MIC value was seen for* E. coli* (0.39, 1.56 *µ*g/mL), followed by* Streptococcus pyogenes* (1.56 *µ*g/mL). The highest MIC value (6.25, 12.5 *µ*g/mL) was recorded for methicillin-resistant* Staphylococcus aureus* (MRSA),* Acinetobacter baumannii*, and* Stenotrophomonas maltophilia*. This study demonstrates, for the first time, the moderate to strong antibacterial activity of miswak extracts against all tested MDR-pathogens. Methanol extract appears to be a potent antimicrobial agent that could be considered as complementary and alternative medicine against resistant pathogens. Further studies on a large number of MDR organisms are necessary to investigate and standardize the inhibitory effect of miswak extracts against these emerging pathogens.

## 1. Introduction

The emergence of multidrug resistant (MDR) human bacterial pathogens during 1990s and more recently the extensively resistant clinical isolates is hampering efforts to control and manage human infections by these organisms [1]. The development of antimicrobial resistance due to misuse of antibiotics is worrisome [2]. Moreover, continuous increase in the global isolation rates of methicillin-resistant* Staphylococcus aureus* (MRSA), methicillin-resistant* Staphylococcus epidermidis* (MRSE), and carbapenem-resistant Gram-negative bacilli clinical isolates poses a serious therapeutic problem because no new antimicrobial agents are currently available for treatment of infected patients [2–4].

Plants are important in human's life and fulfill his every day's needs. They are used as cosmetics, food, flavors, ornamental, and medicine. Medicinal plants have become part of complementary medicine worldwide, because of their potential health benefits. Various plant extract has great potential against infectious agents and can be used for therapeutic purposes [5, 6].

The toothbrush tree,* Salvadora persica* L, also called miswak, belonging to the Salvadoraceae family, is one of the most important ones among 182 species of plants being used as chewing sticks. It has been widely used in many Asian, African, and Middle Eastern countries. The roots, twigs, and stems of this plant have been used for oral hygiene and small miswak sticks have been used as toothpicks for maintaining oral hygiene [7, 8]. It has been reported that the aqueous and methanol extracts of miswak possess various biological properties against organisms considered important for the development of dental plaque and periodontitis [9].

Previous in vitro studies have reported the antibacterial and antifungal effects of miswak on cariogenic bacteria and periodontal pathogens including* Staphylococcus aureus*,* Streptococcus mutans*,* Streptococcus faecalis*,* Streptococcus pyogenes*,* Lactobacillus acidophilus*,* Pseudomonas aeruginosa*,* Aggregatibacter actinomycetemcomitans*,* Porphyromonas gingivalis*,* Haemophilus influenzae*, and* Candida albicans* [10–16]. Moreover, data from controlled clinical studies showed that* Salvadora persica* extract is also an effective antimicrobial agent when utilized clinically as an irrigant in the endodontic treatment of teeth with necrotic pulps [17–20].

Much effort has focused on examining the inhibitory effect of* Salvadora persica* on oral organisms, but information concerning the antibacterial activity of* Salvadora persica* against other human pathogens, particularly MDR isolates, is scarce [14, 15]. Therefore, this study aimed to assess the in vitro antibacterial activities of* Salvadora persica* L. extracts against 10 MDR bacterial clinical isolates (other than oral pathogens) including methicillin-resistant* Staphylococcus aureus* (MRSA), methicillin-resistant* Staphylococcus epidermidis* (MRSE), penicillin-resistant* Streptococcus pyogenes*,* Enterococcus faecalis*, and 6 carbapenem-resistant Gram-negative bacilli:* Escherichia coli*,* Klebsiella pneumoniae*,* Pseudomonas aeruginosa*,* Serratia marcescens*,* Acinetobacter baumannii*, and* Stenotrophomonas maltophilia*.

## 2. Materials and Methods

This in vitro descriptive study was conducted during the period from March to August 2015. A total of 10 pathogenic MDR bacterial strains other than oral pathogens were included in this study.

### 2.1. Bacterial Clinical Isolates

All bacterial strains were isolated from clinical specimens of hospitalized patients with nosocomial infections identified according to the Centers for Disease Control and Prevention/National Healthcare Safety Network (CDC/NHSN) criteria [22]. The selected organisms were MRSA, MRSE, penicillin-resistant* Streptococcus pyogenes*,* Enterococcus faecalis*, and 6 carbapenem-resistant Gram-negative bacilli:* Escherichia coli*,* Klebsiella pneumoniae*,* Pseudomonas aeruginosa*,* Serratia marcescens*,* Acinetobacter baumannii*, and* Stenotrophomonas maltophilia*. All isolates were identified to the species level using standard methods and verified using the VITEK-2 system (Biomérieux, Marcy-l'Etoile, France) according to the manufacturer's instructions. Seven nosocomial strains were isolated from urinary tract infection, two strains were isolated from blood stream infection, and one strain was isolated from ventilator-associated pneumonia. Antimicrobial resistance patterns of all isolates were determined using the reference broth microdilution method following the guidelines of the Clinical and Laboratory Standards Institute (CLSI) [21]. Methicillin resistance among* S. aureus* and* S. epidermidis* strains was identified by PCR amplification of the* mec*A gene as previously described [23]. Carbapenem resistance among Gram-negative bacilli was confirmed using the modified Hodge test following the CLSI guidelines [21]. Multidrug resistance (MDR) of the isolates was identified by resistance to ≥3 of the following antimicrobial classes: penicillins, cephalosporins, aminoglycosides, carbapenems, and quinolones [21].

### 2.2. Miswak Collection

*Salvadora persica* chewing sticks were purchased from the local market of Jazan Province in Saudi Arabia during spring 2015 when plants were flowering. The sticks were washed with distilled water, cut into small species, and allowed to dry at room temperature for 2 weeks. Then, they were grounded to powder using electrical blinder.

### 2.3. Extracts Preparation

Preparation of aqueous and methanol extracts was carried out by mixing 100 g of* Salvadora persica* powder with 1 L of distilled water (for aqueous extract) and 95% methanol (Sigma-Aldrich, St. Louis, USA), with methanol extract for 24 h. The mixture was then filtered using Whatman number 1 filter paper, and the filtrate was then evaporated in vacuum evaporator at 60°C (for aqueous) and 40°C (for methanol). The extracts were stored in sterile bottles and kept frozen at −20°C until further use [24]. Before testing, the miswak extracts were freshly reconstituted in methanol (for methanolic extract) and water (for aqueous extract) at a final concentration of 400 mg/mL which was used to further prepare serial dilutions (400–50 mg/mL).

### 2.4. Inoculum Preparation

All bacterial isolates were grown to the exponential phase in tryptic soy broth (TSB) (Difco Laboratories, Detroit, USA) at 37°C for 18 h. The bacterial growth was estimated as turbidity using spectrophotometer to measure the light absorption of the microbial mass as determined by the optical density readings at 620 nm (OD_620_). Growth was checked every 30 minutes, and the exponential phase of bacterial growth was identified by the increased OD_620_ reading. Then, the inoculum density of each bacterial suspension was adjusted to a final density equivalent to 0.5 McFarland Standard (1.5 × 10^8^ CFU/mL) in sterile saline (0.84% NaCl).

*Antimicrobial Testing*. The antimicrobial activity of miswak extracts was carried out using the agar diffusion and minimal inhibitory concentration (MIC) methods.

#### 2.4.1. Agar Diffusion Method

The antimicrobial testing was performed on Mueller Hinton agar plates (Difco Laboratories) using the agar diffusion method. Briefly, 100 *µ*L of bacterial suspension was spread smoothly on the agar plates. The required numbers of wells, each 3 mm in diameter, were cut out of the agar using a sterile glass capillary ensuring proper distribution of holes in the periphery and one in the center for each agar plate. Then, wells were filled with 50 *µ*L of sterile extract (aqueous or methanol) made from* Salvadora persica* stock solution (400, 200, 100, and 50 mg/mL). This was followed by 2 h preincubation at room temperature for proper diffusion of the plant extract into the media. Then, the plates were incubated at 37°C for 24 h [25]. The mean diameter of complete growth inhibition zone (in mm) was measured without the well's diameter and considered as the inhibition zone. Antibiotic discs (Difco Laboratories, Detroit, USA), 30 *µ*g vancomycin for Gram-positive isolates and 10 *µ*g tobramycin for Gram-negative strains, were used as positive controls, and water (for aqueous extract) or methanol (for methanol extract) was used as negative control. The test for each microorganism was repeated three times to ensure reproducibility. The average zones diameter values from three repeats were taken in determination of the final inhibition zones. This was done to ensure that all inhibition zones within each experiment were obtained under the same experimental conditions.

#### 2.4.2. The Minimal Inhibitory Concentration (MIC)

The MIC of the* Salvadora persica* extracts was determined using the standard microdilution method in 96 multi-well microtiter plates, as previously described [26], with slight modifications. Briefly, the dissolved extracts were first diluted to a concentration of 50 mg/mL, then 50 *µ*L from each of the aqueous and methanol extracts was pipetted into the first well of each microtiter plate row, and 50 *µ*L of TSB was distributed from the 1st to the 12th well of each row. Twofold serial dilution was achieved by transferring 50 *µ*L of scalar dilution from the first to the subsequent wells of each row. The final concentration of the extracts adopted to evaluate antibacterial activity was included from 25 mg/mL to 0.003 mg/mL. Finally, 10 *µ*L of each bacterial suspension was added to each well. Two row lines in each plate were used as controls: one row line with vancomycin as a positive control for Gram-positive isolates and another row line with tobramycin for Gram-negatives strains (in a serial dilution of 32–0.015 *µ*g/mL). Plates were incubated at 37°C for 18–24 h. The lowest concentration at which no turbidity occurred was taken as the MIC value. Plates were analyzed individually to determine MIC and the average MIC values from three repeats were taken in determination of the final MIC values for each extract to ensure accuracy and reproducibility.

## 3. Results

The antibacterial activities of aqueous and methanol extracts of* Salvadora persica* against 10 MDR pathogenic organisms are listed in Table 1. Overall, the 400 mg/mL of miswak extracts was the most effective ones on all strains. The methanol extract of miswak had growth inhibitory effects on the tested pathogens more than aqueous extract. The methanol extract exhibited a stronger antibacterial activity against Gram-negative (3.3–13.6 mm) than Gram-positive (1.8–8.3 mm) bacteria. The highest growth inhibition was recorded against* E. coli* (4.8–13.6 mm), followed by* K. pneumoniae* (4.6–12.7 mm) and* Serratia marcescens* (4.5–12.5 mm).* Streptococcus pyogenes* was the most susceptible one to methanol extract among all Gram-positive pathogens with inhibition zone diameter of 2.2–7.4 mm.

The MIC values of aqueous and methanol extracts are presented in Table 2. The lowest MIC value was seen for* E. coli* (0.39 mg/mL for methanol extract and 1.56 mg/mL for aqueous extract), followed by* Streptococcus pyogenes* (1.56 mg/mL for both extracts). The highest MIC value (12.5 mg/mL for aqueous extract and 6.25 mg/mL for methanol extract) was recorded for MRSA,* Acinetobacter baumannii*, and* Stenotrophomonas maltophilia*.

## 4. Discussion

Antimicrobial resistance has always been a global health concern challenging treatment of human infections caused by MDR-bacterial pathogens [1]. This problem has opened a wide range of research studies investigating the possible use of natural plant extracts in traditional medicine. In the present study, a variety of MDR Gram-positive and Gram-negative bacteria were used in screening antimicrobial activity of aqueous and methanol extracts of* Salvadora persica*.

In this study, the 400 mg/mL of miswak extracts was the most effective one on all pathogens and the methanol extract exhibited a stronger antibacterial activity against Gram-negative than Gram-positive bacteria. These results are in agreement with previous findings from other studies [10, 11, 13, 16, 17]. Contrary to these findings, Al-Bayati and Sulaiman investigated the aqueous and methanol extracts of* Salvadora persica* for antimicrobial activities against seven oral pathogens [12]. In their study, the aqueous extract inhibited all isolated microorganisms and was more efficient than the methanol extract. It is well known that the antimicrobial property of* Salvadora persica* extracts is attributed to the different phytochemical constituents. Mohammed investigated the phytochemical constituents of* Salvadora persica* extracts and revealed the presence of flavonoids, sterols, saponins, tannins, basic alkaloids, and reducing components in methanol extract and saponins, tannins, and reducing components in aqueous extract which could be responsible for the observed antimicrobial property of methanol extract compared with aqueous extract [16]. Sofrata et al. identified a volatile compound: benzyl isothiocyanate (BITC) in* Salvadora persica* extracts [27]. In their study, the BITC exhibited rapid and strong bactericidal effect against Gram-negative bacteria but low effect on Gram-positive bacteria. The authors speculated that BITC might penetrate through the outer bacterial membrane and possibly interfere with the bacterial redox systems and thus hamper the ability of the bacterium to maintain its membrane potential [27].

In this study, the methanol extract had promising MIC values against* E. coli*,* K. pneumoniae*,* P. aeruginosa*, and* S. marcescens* among Gram-negative pathogens and* Streptococcus pyogenes* among Gram-positive bacteria. Previous studies have reported that* Salvadora persica* extracts were effective against* S. aureus*,* Streptococcus mutans*,* Streptococcus pyogenes*,* E. faecalis*, and* P. aeruginosa* [9–13, 16, 17]. However, high MIC values were reported in this study against MRSA,* A. baumannii*, and* S. maltophilia*. To the best of our knowledge, this is the first study investigating the antibacterial activity of miswak extracts against these MDR-pathogens. Our finding is of considerable concern. These emerging pathogens become a significant health problem because of their remarkable ability to innate and acquire resistance to multiple antimicrobial classes and to survive in nosocomial environments [1, 3, 4].

## 5. Conclusion

This study demonstrates, for the first time, the strong to moderate antibacterial activity of methanol and aqueous miswak extracts against all tested MDR-pathogens. However, methanol extract has stronger antibacterial effect, and it appears to be a potent antimicrobial agent that could be considered as complementary and alternative medicines against resistant pathogens. Further in vitro and in vivo studies on a large number of clinical isolates of MRSA,* A. baumannii*, and* S. maltophilia* are necessary to further investigate and standardize the inhibitory effect of miswak extracts against these emerging pathogens.

## Figures and Tables

**Table 1:** The antibacterial activities of aqueous and methanol extracts of miswak against 10 MDR pathogenic organisms.

MDR bacterial isolates^*∗*^	The diameter of inhibition zones (mm)									
	Aqueous extract (mg/mL)				Methanol extract (mg/mL)				Positive control^*∗∗*^	
	400	200	100	50	400	200	100	50	VAN (30 *µ*g)	TOB (10 *µ*g)
MRSA	6.2	5.1	3.8	3.0	8.3	5.1	3.4	2.0	18	NT
MRSE	6.0	4.8	3.2	2.7	7.6	5.8	3.1	1.8	19	NT
*Streptococcus pyogenes*	6.4	4.5	3.5	2.6	7.4	5.4	3.0	2.2	21	NT
*E. faecalis*	6.1	5.2	4.0	3.2	7.8	6.1	5.0	2.9	19	NT
*E. coli*	12.3	9.7	7.2	5.3	13.6	9.2	6.5	4.8	NT	21
*K. pneumonia*	11.8	8.5	7.0	4.3	12.7	8.7	5.8	4.6	NT	20
*P. aeruginosa*	10.5	8.0	5.9	4.0	10.2	8.8	6.2	4.2	NT	17
*S. marcescens*	12.0	9.2	5.6	4.8	12.5	8.1	5.0	4.5	NT	18
*A. baumannii*	8.5	7.3	4.6	3.2	9.8	8.0	5.2	3.5	NT	16
*S. maltophilia*	8.2	7.0	4.8	3.0	9.5	7.6	4.9	3.3	NT	16

^*∗*^MRSA: methicillin-resistant *staphylococcus aureus*, MRSE: methicillin-resistant *staphylococcus epidermidis*.

^*∗∗*^VAN: vancomycin, TOB: tobramycin, and NT: Not tested.

The CLSI zone diameter interpretive criteria for vancomycin (VAN): ≥15 mm: susceptible, 13-14 mm: intermediate, and ≤12 mm: resistant [21].

The CLSI zone diameter interpretive criteria for tobramycin (TOB): ≥17 mm: susceptible, 15-16 mm: intermediate, and ≤14 mm: resistant [21].

**Table 2:** The MIC values of aqueous and methanol extracts of miswak against 10 MDR pathogenic organisms.

MDR bacterial isolates^*∗*^	MIC values (mg/mL)		MIC values (*µ*g/mL)^*∗∗*^	
	Miswak extracts		Positive control	
	Aqueous	Methanol	VAN	TOB
MRSA	12.5	6.25	1.0	NT
MRSE	6.25	3.125	0.5	NT
*Streptococcus pyogenes*	1.56	1.56	0.125	NT
*E. faecalis*	3.125	3.125	0.5	NT
*E. coli*	1.56	0.39	NT	0.25
*K. pneumonia*	3.125	0.781	NT	0.5
*P. aeruginosa*	6.25	1.56	NT	1.0
*S. marcescens*	1.56	1.56	NT	2.0
*A. baumannii*	12.5	6.25	NT	8.0
*S. maltophilia*	12.5	6.25	NT	4.0

^*∗*^MRSA: methicillin-resistant *staphylococcus aureus*, MRSE: methicillin-resistant *staphylococcus epidermidis*.

^*∗∗*^VAN: vancomycin, TOB: tobramycin, and NT: not tested.

The CLSI MIC Interpretive Criteria for vancomycin (VAN): ≤2 *µ*g/mL: Susceptible, 4–8 *µ*g/mL: Intermediate, ≥16 *µ*g/mL: Resistant [21].

The CLSI MIC interpretive criteria for tobramycin (TOB): ≤4 ug/mL: susceptible, 8 *µ*g/mL: intermediate, and ≥16 *µ*g/mL: resistant [21].
